# Data Mining for ICD-10 Admission Diagnoses Preceding Tuberculosis within 1 Year among Non-HIV and Non-Diabetes Patients

**DOI:** 10.3390/tropicalmed7040061

**Published:** 2022-04-14

**Authors:** Ponlagrit Kumwichar, Virasakdi Chongsuvivatwong

**Affiliations:** Department of Epidemiology, Faculty of Medicine, Prince of Songkla University, Kanjanavanich Rd, Kho Hong, Hat Yai District, Songkhla 90110, Thailand; ponlagrit.k@psu.ac.th

**Keywords:** comorbidity, tuberculosis, data mining

## Abstract

Delayed diagnosis of tuberculosis (TB) increases mortality and extends the duration of disease transmission. This study aimed to identify significant ICD-10 admission diagnoses preceding TB. All hospital electronic medical records from fiscal year 2015 to 2020 in the Songkhla Province, Thailand were retrieved. After excluding diabetes and HIV patients, a case-control analysis was performed. Exposures of interest were ICD-10 diagnoses on admissions 1–12 months prior to the visit during which TB was detected. Incident cases of respiratory tuberculosis (A15.0–A16.9) that had been admitted with at least one such exposure were chosen. For every case, controls were retrieved from weekly concurrent OPD patients who had the same 10-year interval of age, sex, and preceding admission and discharge week as the case. The 10 most common comorbidities during hospitalization preceding TB with their relative odds ratios (RORs) and 95% confidence intervals were identified. These included five significant exposures related to lower respiratory infection without adequate TB investigation. Significant RORs ranged from 3.10 (unspecified pneumonia) to 34.69 (hemoptysis). Full TB investigation was not performed due to problems with health insurance. In conclusion, the physicians should be informed about this pitfall, and the insurance system should be revised accordingly.

## 1. Introduction

Tuberculosis (TB) is a chronic infectious disease that is one of the 10 leading causes of death in lower- and middle-income countries [1]. Since TB is an airborne disease, aerosolization of droplet nuclei is the most likely mode of exposure from an infected individual to others [2]. Once a person is infected, TB can affect any body part, particularly the lungs. TB is active if the disease manifests in the affected part. If the immune response inhibits the development of TB and no disease manifestation occurs, the infected person has latent TB infection (LTBI) [3]. LTBI is not contagious, but it can become active TB if the immune response is unable to stop TB from growing [4]. The incubation period of active TB usually ranges from 1 month to 2 years (90% within 1–12 months), after which the chance of developing active TB wanes with time [5]. Additionally, without a potential screening system, by the time that TB is diagnosed, is often too late to prevent transmission [6].

Thailand was one of the 30 countries with the highest TB burden in 2020 [2]. More than 100,000 active TB cases are reported annually [7]. A root cause of this high incidence is delayed TB diagnosis, which is not easily remedied, especially in poor socioeconomic settings [8]. TB can mimic many diseases in the meninges, lungs, and abdomen [9,10,11], thereby leading to misdiagnosis. Moreover, a review of TB admission in 2009 showed that approximately 20% of TB cases were admitted without knowing that the patients had active TB, and TB disease proved fatal for 20% of those without TB detection [12]. For the remainder of the patients, TB was usually detected when the patients were scheduled for follow-up at the outpatient department (OPD). It is hypothesized that missing TB detection at this stage leads to in-hospital and household transmission.

Most previous studies on disease-associated TB were case-control or cohort studies assessing the effect of well-known diseases, such as human immunodeficiency virus (HIV), diabetes mellitus (DM), and other diseases or behavioral factors based on medical conjectures [13,14,15,16,17,18,19,20,21]. In Thailand, TB screening in DM and HIV patients has been practiced, but efforts to detect active TB in the general population are lacking [22,23]. Additional strategies for early diagnosis of active TB, isolation, and initiation of treatment to minimize disease severity are therefore required. Hence, additional knowledge regarding the early diagnosis of TB in Thailand should be specifically assimilated, and the application of this knowledge in poor socioeconomic settings is essential.

Using electronic health records (EHRs), we introduced an approach to identify comorbidities or manifestations that precede TB based on a case-control study to develop a novel policy for early diagnosis of TB. Thus, this study aimed to identify the 10 most commonly hospitalized comorbidities preceding TB disease presentation and measure the significance of these covariates.

## 2. Materials and Methods

### 2.1. Concept and Study Design

The concept of this study is based on a case-control design. Hospitalized comorbidities predicting TB could be more common among subsequent TB cases than among hospital controls who were not subsequently identified with TB disease.

The comorbidities of DM and HIV are well known among practicing physicians to be clinical risk factors for TB [22,23] and any TB disease diagnosis accompanying these comorbidities is less likely to be missed during hospital admission [12]. Therefore, we investigated what other comorbid conditions could predict subsequent active TB.

### 2.2. Data Collection and Retrieval

Relational data from in-patient (IP) and outpatient (OP) electronic medical records from 17 public hospitals (2 tertiary and 15 secondary levels) in Songkhla Province covering the time period April 1, 2015 to October 28, 2020 were deidentified and encrypted for personal information and retrieved from e-claim data of the 12th Regional National Health Security Office (NHSO). Data were previously audited for claim and medical expense information and were collected without missing information on age, sex, service codes, date of services, and ICD-10 diagnoses. Data of patients who lived outside Songkhla Province were excluded. IP and OP records with ICD-10 diagnoses in the categories F, H, O-Q, S, T, and V-Z were eliminated according to the reasons explained in Table 1. Patients without hospital admission history and those aged under 1 year or over 80 years were excluded. The remaining data were used to select cases and controls.

### 2.3. Case Ascertainment

Cases were OP TB patients coded as A15.0–A16.9 from 1 January 2016 to 28 October 2020. Cases without any preceding admission within 1–12 months before the date of diagnosis or with HIV and or DM were excluded. Besides ICD-10 codes, TB diagnosis was confirmed based on available pharmacy data for the allocation of prescribed anti-TB drug treatment regimens for smear-positive patients [24]. Individuals having a culture-negative TB confirmation were excluded.

### 2.4. Control Selection

For every case, controls without HIV or DM were retrieved from weekly concurrent OP who had the same 10-year interval of age, sex, and preceding admission and discharge week as the case. If many admissions could be matched, only the oldest one was selected. Those who had been receiving any anti-TB drugs were excluded.

### 2.5. Matching Ratio and Matching Process

The ratio between cases and controls in each matched set varied depending on the number of cases and available controls for each particular week. The number of controls was limited to 4 per case (1:1, 1:2, 1:3, or 1:4). When more than four eligible controls were available, only four subjects were randomly selected. The looping process for matching was used until all cases were selected. Each case was randomly selected to match with eligible controls. The controls that were chosen could not be matched with the next case.

### 2.6. Outcome

The outcome variable was automatically defined from the subject selection as the TB case (outcome = 1) and control (outcome = 0). 

### 2.7. Exposures

**Figure 1:**
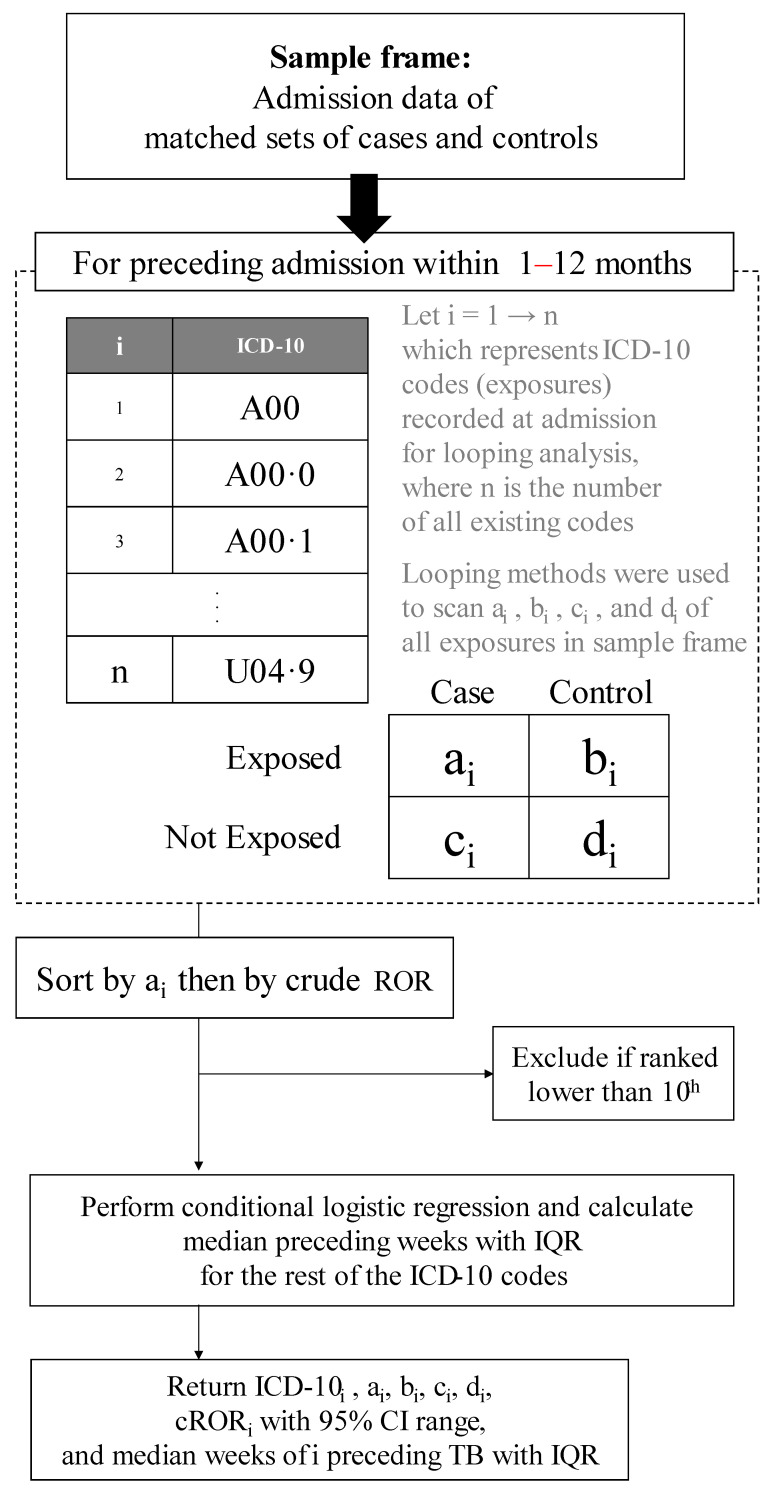
Algorithm to rank the 10 commonly hospitalized comorbidities preceding tuberculosis. CI, confidence interval; cROR, conditional relative odds ratio; IQR, interquartile range; ROR, relative odds ratio; TB, tuberculosis.

The period of available exposure for our exploration was 1 to 12 months before the initial diagnosis week of cases. Since 90% of active TB takes place after this period, we did not include admission beyond 12 months to reduce noise from irrelevant hospitalized comorbidities. The looping method was used to perform a 2 by 2 discordant tables can through all ICD-10 codes to select only the 10 most common ones, as shown in Figure 1. Each of these diseases was used as an exposure variable, one at a time: exposure = 1 for that disease, and 0 otherwise.

### 2.8. Statistical Analysis

Matched sets were constructed based on perfect matching and the age-sex distribution was summarized in tables. The cases and controls who had the same matched variables were placed in the same match set. Due to the matching nature of the study design, the conditional relative odds ratio (cROR) was computed with a 95% confidence interval for each exposure. The preceding period since discharge of each exposure was also computed and reported as its median and interquartile range (IQR). This was repeated until the top 10 diagnoses were analyzed. All analyses were performed using the epiDisplay and tidyverse packages on R language and environment version 4.1.1 (R Core Team (2021). R: A language and environment for statistical computing. R Foundation for Statistical Computing, Vienna, Austria). A *p*-value of ≤0.05 was considered statistically significant. 

### 2.9. Further Exploration for Hospitalized Comorbidities Preceding 

For statistically significant exposures, IP and OP records of all cases were re-reviewed to verify whether and how cases were investigated for bacteriologic evidence of TB.

## 3. Results

### 3.1. Matching Results and Their Characteristics

**Figure 2:**
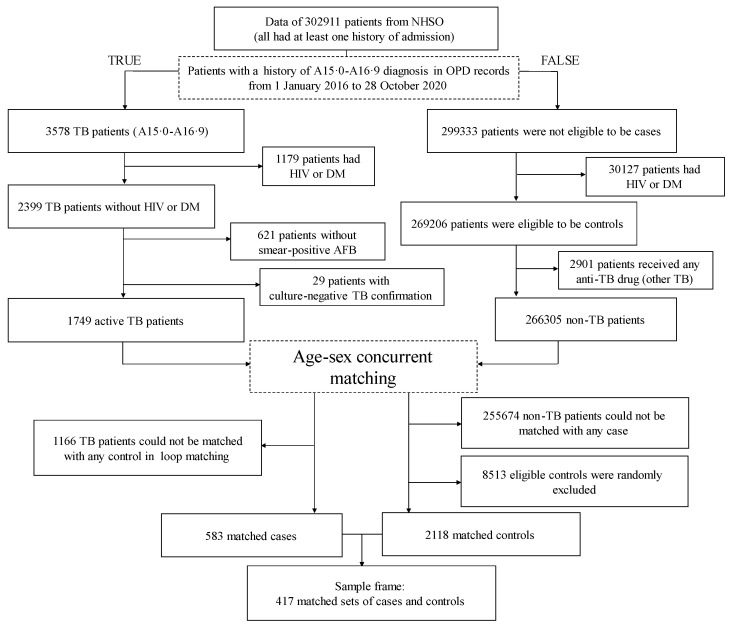
Study flow chart. AFB, acid-fast bacilli; DM, diabetes mellitus; HIV, human immunodeficiency virus; NHSO, National Health Security Office; OPD, outpatient department; TB, tuberculosis.

The numbers of patients included and excluded in the different steps of the case and control selection are shown in Figure 2. Eventually, we identified 417 matched sets (583 cases and 2118 controls). According to Table 2, over two-thirds of the cases were men. TB diagnosis was least common among individuals aged <20 years.

### 3.2. Preceding Admitted Diseases Returned from the Looping Analysis

Table 3 shows the 10 most common exposures preceding TB among patients without HIV and DM. They were J18.9, E87.6, J15.9, E87.1, J90, R04.2, J44.1, J18.1, A09.9, and R50.9, respectively. The significant exposures were unspecified pneumonia (J18.9), unspecified bacterial pneumonia (J15.9), hypo-osmolality and hyponatremia (E87.1), unspecified pleural effusion (J90), hemoptysis (R04.2), and unspecified lobar pneumonia (J18.1). J18.9, J15.9, J90, R04.2, and J18.1 were mutually exclusive of each other. Of note, these 5 diagnoses were principal diagnoses of admission while E87.1 was not a principal diagnosis of any admission. Therefore, we listed the principal diagnoses in Table 4, and 75% of them were pneumonia. Each one had only one principal diagnosis.

### 3.3. Bacteriological Evidence for TB

All admissions with J18.9, J15.9, J90, R04.2, or J18.1 were investigated using sputum AFB. None of them showed a positive result. Neither the cartridge-based nucleic acid amplification test (Gene-Xpert, Cepheid, Sunnyvale, CA, USA) nor TB culture were used as they were not covered by insurance for patients with pneumonia.

For J90, with 10 (58.8%) of 17 cases having thoracentesis, pleural fluid samples were tested for AFB and all of them were negative. None of the fluid samples underwent adenosine deaminase (ADA) testing. Other profile data of the pleural fluid were not available. 

## 4. Discussion

After excluding HIV, DM, and known LTBI, only one-quarter of the TB cases could be matched for this data mining. The 10 most common comorbidities preceding TB were mostly related to lower respiratory problems. All cases with lower respiratory symptoms had negative sputum AFB results. Additionally, hyponatremia was the most common accompanying diagnosis with pneumonia.

Our data re-confirmed the inadequate sensitivity of the sputum AFB test to detect TB among the hospitalized patients who manifested with lower respiratory problems. Pneumonic TB is a common TB manifestation; however, early TB is usually misdiagnosed especially without bacteriological evidence [25]. Without cavity formation, TB may grow slowly, resulting in a small number of *Mycobacterium tuberculosis* that cannot be detected by AFB [26].

Pleural effusion is an even more difficult clinical entity regarding obtaining its etiologic agent. A low yield for AFB and even Gene-Xpert is well known [27,28]. With the optimal cut-off point (>40 IU.L^−1^), the ADA test is more useful, with both the sensitivity and specificity being 80–100% [29]. However, this test is usually performed at a referral hospital along with other investigations. At rural hospitals, where TB is common, these suspected cases are either referred or discharged and followed up. A delay in TB diagnosis is therefore often inevitable.

Hemoptysis has been classically described as a manifestation of pulmonary TB [30]. The sensitivity of AFB to detect TB with hemoptysis is approximately 30% [31]. The finding that some patients with hemoptysis and negative AFB were later confirmed to have TB warrants further investigation.

The role of hyponatremia in the early manifestation of TB should be further investigated. Its presence may be a clue for the diagnosis of TB. A plausible mechanism may include a syndrome of inappropriate antidiuretic hormone secretion [32,33] and poor intake, which are associated with active TB [34].

Although universal health coverage in Thailand covers full TB investigation for suspects, hospitalized lower respiratory infections have not yet been included. A lack of awareness of TB’s ability to mimic these diseases combined with the lack of support regarding insurance has increased the pitfalls of missing TB in hospitalized patients. The problems should therefore be solved on both sides. 

Our data may not have been adequate for the detection of small effect sizes of many comorbidities. The exclusion of HIV and DM patients may have eliminated other significant comorbidities. Our analysis did not take into account confounders because socioeconomic variables were not available in the current EHRs. ROR in this study compared the exposure of interest with other exposures. Hence, ROR could not be interpreted as a conventional odds ratio.

This study was conducted in Songkhla Province, a southern province of Thailand. These findings should be interpreted with caution before being applied to other settings.

## 5. Conclusions

The most common hospitalized comorbidities preceding TB were related to lower respiratory infection. This suggests that TB was missed in diagnosis as it often mimics other lower respiratory infections. In lower- and middle-income countries, physicians treating patients with lower respiratory infections should be reminded about this pitfall. Full investigation for possible pulmonary TB was not performed by the health system due to problems with health insurance. Therefore, physicians should be informed about this pitfall, and the insurance system should be revised accordingly.

## Figures and Tables

**Table 1:** Categories for the exclusion of irrelevant diseases.

Category	Decode	Reasons
F	Mental, behavioral, and neurodevelopmental disorders	The cases are usually passed to a specific department or psychiatric hospital; thus, the patients are not proper controls.
O, P, Q	Pregnancy, childbirth, and puerperium Certain conditions originating in the perinatal period	The diseases/conditions in these categories are rarely related to TB.
S, T, V, W, X, Y	External causes of morbidity and mortality	Almost all cases belong to the emergency department, but ED data are also recorded in the OPD database
Z	Factors influencing health status and contact with health services	These are not codes for hospitalized comorbidities

**Table 2:** Age and sex distribution of the matched sets.

Characteristics	TB Case	Concurrent Matched Controls	Concurrent Matched Sets
Age group, *n* (%)			
**Male**			
1–10	30 (5.1)	119 (5.6)	26 (6.2)
11–20	16 (2.7)	64 (3.0)	13 (3.1)
21–30	77 (13.2)	291 (13.7)	51 (12.2)
31–40	74 (12.7)	281 (13.3)	47 (11.3)
41–50	75 (12.9)	251 (11.9)	53 (12.7)
51–60	67 (11.5)	219 (10.3)	49 (11.8)
61–70	43 (7.4)	141 (6.7)	28 (6.7)
71–80	36 (6.2)	126 (6.0)	22 (5.3)
**Female**			
1–10	34 (5.8)	136 (6.4)	26 (6.2)
11–20	14 (2.4)	51 (2.4)	13 (3.1)
21–30	29 (5.0)	112 (5.3)	23 (5.5)
31–40	32 (5.5)	121 (5.7)	26 (6.2)
41–50	16 (2.7)	58 (2.7)	12 (3.0)
51–60	15 (2.6)	57 (2.7)	11 (2.6)
61–70	11 (1.9)	40 (1.9)	8 (1.9)
71–80	14 (2.4)	51 (2.4)	9 (2.2)

**Table 3:** Ten most frequent hospitalized comorbidities preceding tuberculosis.

ICD-10	Diagnosis	Case		Control		Median Preceding Weeks (IQR)	Conditional ROR (95% CI)	
		Exposed (a)	Not Exposed (b)	Exposed (c)	Not Exposed (d)			*p*-Value
J18.9	Unspecified pneumonia	33	550	39	2079	31 (19, 42)	3.10 (1.91, 4.98)	<0.001
E87.6	Hypokalemia	30	553	99	2019	30 (17, 40)	1.04 (0.68, 1.61)	0.854
J15.9	Unspecified bacterial pneumonia	21	562	35	2083	23 (14, 33)	2.13 (1.21, 3.74)	0.008
E87.1	hypo-osmolality and hyponatremia ^1^	20	563	33	2085	25 (18, 37)	2.14 (1.18, 3.87)	0.012
J90	Unclassified pleural effusion	17	566	9	2109	30 (21, 46)	6.15 (2.68, 14.14)	<0.001 *
R04.2	Hemoptysis	10	573	1	2117	30 (13, 36)	34.69 (4.40, 273.39)	<0.001 *
J44.1	Unspecified COPD with acute exacerbation	10	573	21	2097	27 (17, 39)	1.63 (0.75, 3.55)	0.215
J18.1	Unspecified lobar pneumonia	9	574	6	2112	24 (20, 34)	6.19 (2.05, 18.77)	0.001
A09.9	Unspecified gastroenteritis and colitis	9	574	50	2068	29 (21, 41)	0.63 (0.31, 1.30)	0.212
R50.9	Unspecified fever	8	575	35	2083	33 (15, 42)	0.85 (0.39, 1.86)	0.690

^1^ The hospitalized comorbidities that were not the principal diagnosis, and were not mutually exclusive of any of the 10 common comorbidities due to hospitalization. COPD, chronic obstructive pulmonary disease. * A *p*-value less than 0.05 is statistically significant.

**Table 4:** Principal diagnoses accompanied by E87.1 among the TB cases.

ICD-10	Principal Diagnosis of the Cases Admitted with E87.1	Frequency *n* (%)
J18.9	Unspecified pneumonia	12 (60)
R50.9	Unspecified fever	3 (15)
J15.9	Unspecified bacterial pneumonia	2 (10)
C34.9	Malignant neoplasm of bronchus or lung	2 (10)
J18.1	Unspecified lobar pneumonia	1 (5)

## Data Availability

The data of the sample frame and codes used in the analysis are available in a GitHub repository. The full data sets of all patients in Songkhla Province could not be shared according to PDPA. For the GitHub repository of this study see at https://github.com/ponlagrit/tb_data_mining (accessed on 31 March 2022).
